# Enlargement of Lymphatic Glands about the Bifurcation of the Trachea and Bronchial Tubes, Obstructing Respiration

**Published:** 1866-12

**Authors:** C. Cushing

**Affiliations:** Turner, Ill.


*Enlargement of Lymphatic Glands about the Bifurcation of the Trachea and Bronchial Tubes Obstructing Respiration.* By C. CUSHING, M. D., Turner, Ill.

On December 28th, 1865, I was called to see an infant, four months of age, affected with a slight cough and difficulty of breathing, and what the old ladies call "snuffles." The child seemed fleshy and well nourished, and as its mother had no breast-milk, it was fed from a bottle. I left some medicine and the child improved somewhat, but in about ten days got worse again, distressing dyspnœa occurring in spasms, simulating asthma, being the most prominent symptoms.

Owing to the extreme youth of the child, and to its struggles and cries, the results of auscultation and percussion were unsatisfactory and threw no light on the case. The dyspnœa was worse at irregular intervals, the child breathing most easily when asleep. Various modes of treatment were instituted by myself and by two other physicians, but nothing seemed of any avail except the warm bath, which gave temporary relief. I treated the case under the belief that it was a spasmodic nervous constriction of the trachea or bronchial tubes. One of the other physicians pronounced it dropsy of the heart; and the other said "wind on the stomach."

The child died about the middle of April, 1866, and I was called upon to make a post-mortem examination: it had lost some flesh since I had seen it in February, but it was not emaciated. All the internal organs were in a healthy condition except the lungs and the contiguous lymphatic glands.

There was extensive tuberculous deposit throughout both lungs, some of the tubercles having already commenced to soften. The tubercles were from the size of a millet seed to that of small peas.

The most interesting pathological conditions, however, were in the enormously enlarged lymphatic glands, situated about the bifurcation of the trachea: there were quite a number of these, some of them an inch in diameter, apparently filled with tuberculous matter.

Now it seemed evident to me that the difficulty of breathing was caused, in a great measure, by the pressure of the enlarged glands upon the trachea and bronchial tubes lessening their calibre, and that the nervous symptoms were caused by the pressure of the enlarged glands upon the pneumogastric nerves, causing spasmodic nervous constriction, more or less, of the whole lung. The trachea, at its upper part, and the larynx were in a perfectly healthy condition.

The father and mother were healthy people, but two children had died out of the family with pulmonary tuberculosis.

---

*Mortality of First Labors.*—Dr. Duncan, in the Edinburgh *Medical Journal*, declares, on the authority of statistics, that the mortality of first labors, and of puerperal fever following first labors, is about twice the mortality attending all subsequent labors collectively; and that after the ninth labor, the mortality increases with the number.

